# Heart Rate Threshold Settings for Ventricular Tachycardia in Patients with Wearable Cardioverter-defibrillators

**DOI:** 10.19102/icrm.2025.17022

**Published:** 2026-02-15

**Authors:** Steve Ringquist, Nicole R. Bianco, Patricia Tung

**Affiliations:** 1 ZOLL CMS, Pittsburgh, PA, USA; 2 Beth Israel Deaconess Medical Center, Boston, MA, USA

**Keywords:** Cardiac defibrillator, cardiovert, ventricular tachycardia, wearable cardioverter-defibrillator

## Abstract

The wearable cardioverter-defibrillator (WCD) uses programmable heart rate (HR) and detection time to determine the need for shock delivery, but the optimal ventricular tachycardia (VT) HR detection zone is unknown. The LifeVest™ (ZOLL Medical, Chelmsford, MA, USA) WCD has a default VT detection rate of 150 bpm. We found that 15% of appropriate shocks among WCD recipients were delivered for VT 150–170 bpm and that 30% of these patients experienced loss of consciousness. Based on these findings, a programmed VT detection threshold of 150 bpm should be considered.

## Introduction

The wearable cardioverter-defibrillator (WCD) is indicated for patients at high risk of sudden arrhythmic death due to recent myocardial infarction or newly diagnosed heart failure and low ejection fraction.^1^ Similar to implantable cardioverter-defibrillators (ICDs), the WCD uses programmable heart rate (HR) and detection time to determine the need for shock delivery. Hemodynamic tolerance of ventricular tachycardia (VT) can vary and is frequently determined by the HR during VT.^2^ Primary-prevention ICDs are typically programmed with a VT detection rate of 170 bpm based on published studies.^3^ However, optimal programming of the VT detection zone in WCD is unknown. Of the two commercially available WCDs in the United States (US), the LifeVest™ (ZOLL Medical, Chelmsford, MA, USA) has a default VT detection rate of 150 bpm, while the ASSURE® (Kestra Medical Technologies Inc., Kirkland, WA, USA) uses a rate of 170 bpm.^4^ We conducted an analysis of appropriate shocks among WCD recipients to determine the frequency and clinical outcomes of shocks for VT 150–170 bpm.

## Methods

We performed a retrospective, cases-only analysis of consecutive US adults who received an appropriate shock from a LifeVest™ WCD during January 1, 2021 to December 31, 2022. All data were collected from the ZOLL Medical registry of US LifeVest™ prescriptions with the following default settings: (1) VT detection at 150 bpm with shock delivery at 60 s and (2) ventricular fibrillation (VF) detection at 200 bpm with shock delivery at 25 s. Shocks were classified as slow VT, defined by an HR between 150 and 170 bpm at onset (VT_150–170_), and fast VT, defined by VT rates of >170 bpm at onset (VT_>170_). There was no control group in this case series, as data regarding the number of inappropriate shocks and the total number of patients receiving the LifeVest™ WCD were not available. All patients provided consent for the use of de-identified data for research at the time of prescription. Given the use of de-identified data, institutional review board approval was not required.

The indication for WCD prescription was collected from the medical order form and the International Classification of Diseases, Tenth Revision, codes. HR, shock, and response button (which delays shock) use data were collected. WCD use was determined from device data during a period of 90 days from the first day of wear. Loss of consciousness (LOC) and survival, defined by being alive 24 h post-event, were determined using information gathered from patients, family, or physicians that was recorded on the medical form. Data were analyzed using the chi-squared test, Student’s *t* test, or the Wilcoxon test as appropriate. Results were reported as means with standard deviation values or medians with interquartile range values. Significance was defined by *P* < .05.

## Results

During the study period, 676 patients received an appropriate shock for VT. The indications for WCD prescription are presented in **Tables 1A–C**. Eight percent of the patients were prescribed the WCD for secondary-prevention indications (cardiac arrest/sustained VT), 5% were prescribed the device for ICD explant (prior history of cardiac arrest unknown), and the remainder were prescribed the device for primary-prevention indications (87%). Of the 676 patients who received an appropriate shock, 103 (15%) received therapy for VT_150–170_, while 573 (85%) received a shock for VT_>170_. Individuals with VT_150–170_ were older and more likely to have heart failure, while individuals with VT_>170_ were more likely to have ischemic cardiomyopathy. The baseline characteristics of patients treated for slow and fast VT are summarized in **Table 1A**. Initial shock therapy occurred, on average, on day 24 for both groups. HR stability and distribution from VT onset to shock delivery were normally distributed and centered at no change, with no change in VT HR from onset to shock seen in most cases **(Table 1B)**. LOC was significantly more likely in patients with VT_>170_ when compared to patients with VT_150–170_ (50% vs. 30%; *P* < .01). Patients with slow VT were more likely to demonstrate response button use compared to patients with fast VT_>170_ (36% vs. 24%; *P* < .05). No significant difference was observed in first shock conversion success or survival at 24 h between the VT_150–170_ and VT_>170_ groups **(Table 1C)**.

**Table 1A:** Summary of Baseline Characteristics

	VT_150–170_ (n = 103)	VT_>170_ (n = 573)	*P* Value
Demographics			
Age, years, mean ± SD	71 ± 12	67 ± 11	**≤.01**
Male, n (%)	78 (76%)	415 (72%)	.55
Reason for WCD prescription, n (%)			
ICD explant	5 (5%)	28 (5%)	1
Ischemic cardiomyopathy	43 (42%)	313 (55%)	**.02**
Dilated cardiomyopathy	34 (33%)	177 (31%)	.76
Cardiac arrest/sustained VT	11 (11%)	42 (7%)	.33
Heart failure	10 (10%)	13 (2%)	**≤.001**

*Abbreviations:* ICD, implantable cardioverter-defibrillator; SD, standard deviation; VT, ventricular tachycardia; WCD, wearable cardioverter-defibrillator. Bold *P* values are statistically significant.

**Table 1B:** Shock Metrics and Outcomes

	VT_150–170_ (n = 103)	VT_>170_ (n = 573)	*P* Value
Shock event metrics			
Day of shock, mean ± SD	24 ± 22	24 ± 23	.91
Response button use, n (%)	37 (36%)	138 (24%)	**.01**
Patients with no change (or decrease) in VT heart rate prior to therapy, n (%)	64 (62%)	388 (68%)	.32
VT duration, s, median (IQR)			
Without response button use	67 (62–68)	33 (32–34)	**<.001**
With response button use	117 (84–208)	83 (61–140)	**.03**

*Abbreviations:* IQR, interquartile range; SD, standard deviation; VT, ventricular tachycardia. Bold *P* values are statistically significant.

**Table 1C:** Therapy Outcomes

Clinical Outcomes, n (%)	VT_150–170_ (n = 103)	VT_>170_ (n = 573)	*P* Value
LOC	31 (30%)	286 (50%)	**≤.001**
Fall	11 (11%)	55 (10%)	.87
Shock conversion	97 (94%)	544 (95%)	.94

*Abbreviations:* LOC, loss of consciousness; VT, ventricular tachycardia. Bold *P* values are statistically significant.

## Discussion

In this first study examining the incidence of slow VT in patients with WCDs, we found that 15% of appropriate shocks were delivered for VT_150–170_, and one-third of these patients experienced LOC. Although the absence of a control group in this study makes it difficult to fully interpret these findings, they can be examined within the context of previously published data of WCD recipients, which suggest a 1.7% rate of sustained VT or VF in this population.^1^ Additional studies found a 3% rate of appropriate therapy among WCD recipients with ischemic cardiomyopathy and a 1%–6% rate among those with nonischemic cardiomyopathy.^5,6^

Several studies have investigated the frequency and clinical impact of slow VT among primary-prevention ICD recipients. The incidence of slow VT in ICD patients was variable, ranging from 30% in one study, which defined slow VT as 101–148 bpm, to 6% in another study, which categorized the slow VT zone as 130–186 bpm.^7,8^ These episodes were successfully treated with antitachycardia pacing and were generally not associated with syncope or presyncope.

A VT detection zone of 170 bpm has been determined to minimize inappropriate therapies while enabling effective treatment of VT among primary-prevention ICD recipients.^3^ While VT HR detection >170 bpm among WCD recipients could decrease the number of inappropriate shocks, it may also prevent or delay treatment of clinically important arrhythmias in a significant proportion of WCD candidates.

## Conclusion

Based on our findings, a programmed VT detection threshold of 150 bpm should be considered for WCD recipients.
